# Retraction: miR-26a prevents neural stem cells from apoptosis via β-catenin signaling pathway in cardiac arrest-induced brain damage

**DOI:** 10.1042/BSR-20181635_RET

**Published:** 2021-05-04

**Keywords:** β-catenin, GSK-3β, miR-26a, NSCs and CA

This Retraction follows an Expression of Concern relating to this article previously published by Portland Press.

This article is being retracted from *Bioscience Reports* at the request of the authors following receipt of a notification from a reader, alerting the authors to a row of duplicated images with differing contrasts in Figure 2A.

The authors are unable to correct the article and wish to retract the article. The Editor-in-Chief and Editorial Board agree with the retraction.

